# Neuropathology of *FMR1*-premutation carriers presenting with dementia and neuropsychiatric symptoms

**DOI:** 10.1093/braincomms/fcab007

**Published:** 2021-01-27

**Authors:** Anke A Dijkstra, Saif N Haify, Niek A Verwey, Niels D Prins, Esmay C van der Toorn, Annemieke J M Rozemuller, Marianna Bugiani, Wilfred F A den Dunnen, Peter K Todd, Nicolas Charlet-Berguerand, Rob Willemsen, Renate K Hukema, Jeroen J M Hoozemans

**Affiliations:** 1 Department of Pathology, Amsterdam Neuroscience, Amsterdam University Medical Centre, Amsterdam, The Netherlands; 2 Department of Clinical Genetics, Erasmus MC, Rotterdam, The Netherlands; 3 Department of Neurology, Medisch Centrum Leeuwarden, Leeuwarden, The Netherlands; 4 Department of Neurology, Alzheimer Center, VU University Medical Center, Amsterdam Neuroscience, The Netherlands; 5 Brain Research Center, Amsterdam, The Netherlands; 6 Department of Pathology and Medical Biology, University Medical Center Groningen, Groningen, The Netherlands; 7 Department of Neurology, University of Michigan, Ann Arbor, MI, USA; 8 Department of Veterans Affairs, Ann Arbor Healthcare System, Ann Arbor, MI, USA; 9 Institut de Génétique et de Biologie Moléculaire et Cellulaire (IGBMC), INSERM U964, CNRS UMR7104, University of Strasbourg, 67400, Illkirch, France; 10 Department of Health Care Studies, Rotterdam University of Applied Sciences, Rotterdam, The Netherlands

**Keywords:** FMR1-premutation, FXAND, FXTAS, neuropathology, nuclear inclusions

## Abstract

CGG repeat expansions within the premutation range (55–200) of the *FMR1* gene can lead to Fragile X-associated tremor/ataxia syndrome and Fragile X-associated neuropsychiatric disorders. These CGG repeats are translated into a toxic polyglycine-containing protein, FMRpolyG. Pathology of Fragile X-associated tremor/ataxia syndrome and Fragile X-associated neuropsychiatric disorders comprises FMRpolyG- and p62-positive intranuclear inclusions. Diagnosing a *FMR1*-premutation carrier remains challenging, as the clinical features overlap with other neurodegenerative diseases. Here, we describe two male cases with Fragile X-associated neuropsychiatric disorders-related symptoms and mild movement disturbances and novel pathological features that can attribute to the variable phenotype. Macroscopically, both donors did not show characteristic white matter lesions on MRI; however, vascular infarcts in cortical- and sub-cortical regions were identified. Immunohistochemistry analyses revealed a high number of FMRpolyG intranuclear inclusions throughout the brain, which were also positive for p62. Importantly, we identified a novel pathological vascular phenotype with inclusions present in pericytes and endothelial cells. Although these results need to be confirmed in more cases, we propose that these vascular lesions in the brain could contribute to the complex symptomology of *FMR1*-premutation carriers. Overall, our report suggests that Fragile X-associated tremor/ataxia syndrome and Fragile X-associated neuropsychiatric disorders may present diverse clinical involvements resembling other types of dementia, and in the absence of genetic testing, FMRpolyG can be used post-mortem to identify premutation carriers.

## Teaser

*FMR1*-premutation carriers can present clinically with a range of symptoms, including neuropsychiatric symptoms. Pathologically, the nuclear inclusions are positive for FMRpolyG and in the donors discussed here, also present in the vasculature. This has not been described before and this finding possibly contributes to the complex phenotype.

## Graphical Abstract

**Graphical Abstract:**
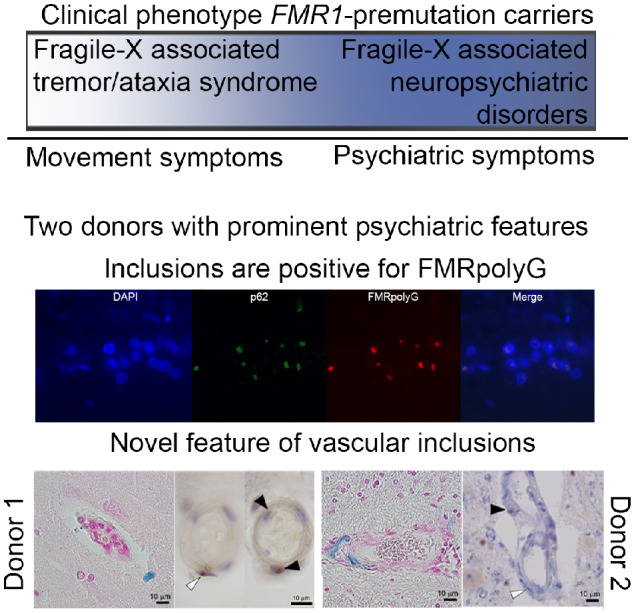

## Introduction

The Fragile X mental retardation 1 (*FMR1*) gene contains a CGG dynamic trinucleotide repeat sequence. Within the general population, this number of CGG repeats is present under 55 repeats (Hagerman and Hagerman, 2015). Expansions of the repeat sequence to more than 200 CGG repeats result in the absence of the *FMR1* protein, and in the Fragile X syndrome (Murray *et al.*, 1997). Smaller expansions (55–200 CGG repeats) are in the premutation range (Brouwer *et al.*, 2009) and can lead to Fragile X-associated diseases, including the neurodegenerative diseases Fragile X-associated tremor/ataxia syndrome (FXTAS) (Berry-Kravis *et al.*, 2007a) and the Fragile X-associated neuropsychiatric disorders (FXAND) (Hagerman *et al.*, 2018). CGG-repeats in the premutation range are transcribed and translated into a toxic polyglycine-containing protein, named FMRpolyG protein (Todd *et al.*, 2013). FMRpolyG is considered to play a major role in the pathogenesis of FXTAS (Sellier *et al.*, 2017; Boivin *et al.*, 2018).

The FXTAS phenotype is found in 30% of male carriers and 11–18% of female carriers of the *FMR1*-premutation and is characterized primarily by intention tremors and cerebellar gait ataxia, and in severe cases accompanied by executive function, memory deficits and Parkinsonism (Jacquemont, 2004; Leehey *et al.*, 2016; Robertson *et al.*, 2016). The onset of the motor symptoms may present after 50 years of age, with longer expansions correlating with an earlier onset and more severe disease (Tassone *et al.*, 2007). However, additional clinical features have been recently described in premutation carriers that include anxiety and depression, which has led to the recent identification of FXAND, encompassing a large group of premutation carriers presented with neuropsychiatric symptoms (Hagerman *et al.*, 2018). Consequently, diagnosing a *FMR1*-premutation carrier remains challenging, as the clinical features may overlap with other neurodegenerative diseases (Grigsby *et al.*, 2007; Seritan *et al.*, 2008). A genetic screening for CGG expansion can provide the conclusive answer, especially when a grandchild or child is diagnosed with a full mutation, leading to the Fragile X syndrome (Young, 1993).

The pathology of an *FMR1*-premutation includes p62-positive and mostly solitary, spherical intranuclear inclusions, present in both neurons and astrocytes and found in broad distribution throughout the brain and brainstem (Greco *et al.*, 2006; Tassone *et al.*, 2012). Post-mortem studies in FXTAS donors also revealed cerebellar and cerebral white matter abnormalities with inclusions in astrocytes, particularly evident in sub-cortical cerebral white matter (Greco *et al.*, 2006). This is consistent with MRI studies that reveal global brain atrophy and white matter signal changes with characteristic feature middle cerebral peduncle presents in about 40% of the patients with FXTAS (Famula *et al.*, 2018). Here, we describe novel pathological features of two cases with a *FMR1-*premutation and a clinical profile of FXAND with mild movement impairments. Our study supports recent studies, showing that testing for FMRpolyG immunoreactivity can confirm the presence of the *FMR1-*premutation (Todd *et al.*, 2013; Krans *et al.*, 2019).

## Materials and methods

### Magnetic resonance imaging

Donors were scanned on a 3T whole-body magnetic resonance system (General Electric, Milwaukee, WI). The protocol included a 3D T1-weighted fast spoiled gradient echo sequence for volumetric measurements and for Donor 2, a 3D T2-weighted fluid-attenuated inversion recovery sequence for white matter lesion segmentation.

### Post-mortem tissue

Post-mortem tissue was obtained through the Netherlands Brain Bank (NBB) and the department of Pathology, University Medical Center Groningen, The Netherlands. Tissue of a spinocerebellar ataxia donor type 3 (SCA3) was obtained through the NBB and used as a negative control for FMRpolyG. Tissue was fixed in 4% PFA for 4 weeks and dissected in 24 diagnostic regions. Sections of 8 μm of each region were cut and stained using haematoxylin and Eosin (H&E) and Perls staining for iron deposits. Donors were routinely immunostained for amyloid-beta (1:800; mouse, clone IC16 kind gift of Dr Korth, Heinrich Heine University, Düsseldorf, Germany), tau (1:800; mouse, clone AT8, Thermo Fisher Scientific, MA, USA), alpha-synuclein (1:1000; mouse, Zymed; Thermo Fisher Scientific, Bleiswijk, The Netherlands), TDP-43 (pTDP43, 1:8000; mouse, clone 11‐9, Cosmo Bio, Japan) and polyQ (1:800; mouse, clone 5TF1-1C2, Merck Millipore, Burlington, WI, USA).

### Immunohistochemical procedures

Sections were deparaffinized and then incubated in 0.3% H_2_O_2_ in phosphate buffer saline (pH = 7.4) for 30 min. Sections were then washed with phosphate buffer saline (3 × 5 min) and antigen retrieval was performed in citrate buffer (pH = 6.0) using an autoclave (121°C for 5 min). After washing, sections were incubated in primary antibody (p62, 1:1000; mouse, clone 3/P62 lck ligand, BD Transduction Laboratories, San Jose, CA, USA) FMRpolyG (NTF1), 1:200; Rabbit, antibody developed by Peter Todd; FMRpolyG (1C5), 1:500; mouse developed by Nicolas Charlet-Berguerand; for 1 h at room temperature. After washing, sections were incubated with HRP-labelled Envision (K5007; DAKO, Glostrup, Denmark), washed and visualization of immunostaining was performed with chromogen 3,3′-diaminobenzidine (K5007; DAKO). Finally, sections were counterstained with haematoxylin, dehydrated and cover slipped with Quick-D (Klinipath, Duiven, The Netherlands). Negative controls were included by omitting the primary antibody and showed no immunoreactivity.

### Double-labelling immunofluorescence

For double-immunofluorescence, sections were incubated for 1 h with a combination of primary antibodies; p62 (1:1000) and FMRpolyG (NTF1) (1:200). Subsequently, sections were incubated with fluorescent-probed secondary antibodies 1:250 (1 h); Goat Anti-Rabbit-594 (Invitrogen, Carlsbad, CA, USA), and Goat Anti-Mouse 488 (Life Technologies, Carlsbad, CA, USA). Auto-fluorescence was blocked with 0.2% Sudan Black for 5 min at room temperature and mounted with DAPI Fluoromount G (Southern Biotech, AL, USA).

### Statistical analysis

No analyses were performed.

### Data availability

Data are available upon request.

## Results

### Clinical profile

#### Donor 1

At the age of 57 years, the patient complained about pain in his legs and subsequently polyneuropathy was diagnosed. At 59 years, he was seen again at the neurological outpatient clinic due to a minor stroke. Besides the sub-acute stroke-related neurological signs, the patient complained about progressive cognitive problems (memory and slowness). Additionally, behavioural changes (apathic and passive) were mentioned. The patient spent whole days just listening to music and was easily agitated and not co-operative. Neurological examination showed several signs of Parkinsonism. Initially, vascular dementia/vascular Parkinsonism or Parkinson’s disease were mentioned as diagnosis. The MRI showed mild vascular changes, and vascular dementia seemed unlikely. As disease progressed, other symptoms became more apparent such as executive problems, increased cognitive decline but also significant bradyphrenia. Clinically, the patient was demented and atypical Alzheimer’s disease (AD) and frontotemporal dementia were added to the differential diagnosis. Biomarkers did not provide any indication for AD. Additionally, an FDG-PET scan was performed, showing a pattern which partially suited corticobasal degeneration or less likely progressive supranuclear palsy (PSP). However, corticobasal degeneration or PSP were clinically less evident, since dementia with significant frontal characteristics was pronounced. The patient showed increased psychiatric problems, including aggressiveness towards partner, apathy and compulsive behaviour. Due to an acute unsustainable situation at home, the patient was taken into a psychiatric hospital where he committed suicide at the age of 62 years. After autopsy, the donor presented intranuclear inclusion pathology. FMRpolyG immunohistochemistry was positive and subsequent genetic testing revealed a 107 CGG repeat within the *FMR1* gene.

#### Donor 2

**Figure 1:**
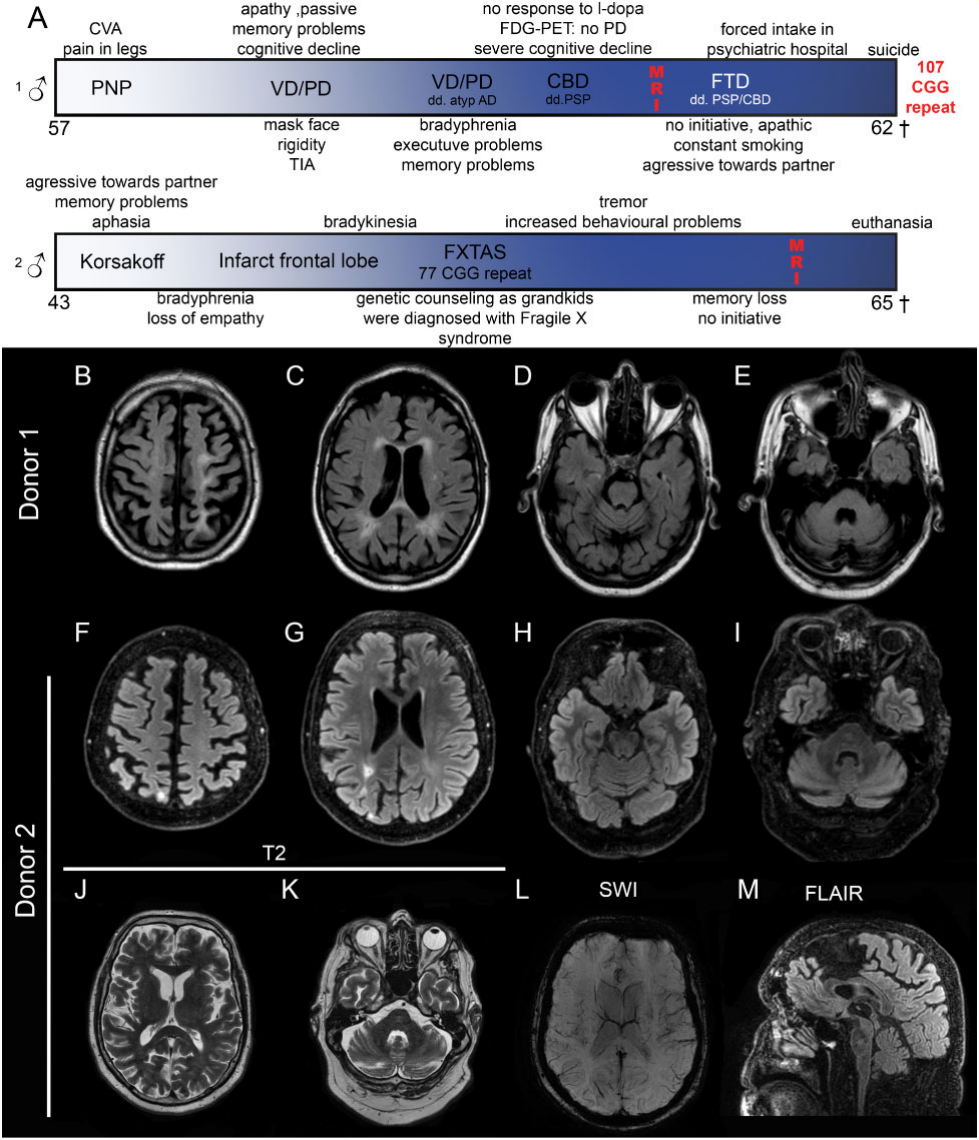
**Clinical course and imaging results of Donors 1 and 2.** Clinical course (**A**) shows moment in time of MRI. General mild atrophy is observed (**B**, **F**). Ventricles are slightly dilated and Donor 1 shows periventricular white matter lesions (**C**), Donor 2 shows an increased signal due to an infarct (**G**). Slight cerebellar atrophy can be seen in both donors (**D**, **E**, **H**, **I**). Enlargement of the fourth ventricle, but no FXTAS-characteristic middle cerebral peduncle sign can be seen. T2-fluid-attenuated inversion recovery of Donor 2 does not show increased insular and middle cerebral peduncle signal intensities (**J**, **K**), no abnormalities in vasculature (**K**) and no thinning of the corpus callosum (**M**). CVA, cerebrovascular accident; PNP, polyneuropathy; VD, vascular dementia; PD, Parkinson’s disease; AD, Alzheimer’s disease; CBD, corticobasal degeneration; FTD, frontotemporal dementia; PSP, progressive supranuclear palsy.

At the age of 43 years, this patient came to the memory clinic with memory problems and aphasia, whereas his partner noted that he became aggressive towards her. He showed signs of social anxiety and suffered from chronic depression. He was initially diagnosed with Korsakoff’s syndrome based on earlier excessive alcohol consumption and the early onset of the symptoms. At 51 years, he complained of memory loss and concentration problems and character changes. At 53 years, he suffered from cardiac palpitations and cramps in his fingers, but no cause was found. He had visual hallucinations, in which he saw persons and felt drawn towards the cemetery. A year later, he experienced a change in gait, and dragged his feet. At 55 years, he had a major stroke in the right occipital region, reporting loss of strength and co-ordination in his left arm and leg, followed by a spontaneous recovery. A year later, he suffered from falling and increased bradykinesia. He had a light tremor in both hands, started snoring and developed sleep apnoea. Around this time, his grandchildren were born and diagnosed with Fragile X-syndrome. Genetic testing revealed a 77 CGG repeat in the *FMR1* gene, and his initial diagnosis of Korsakoff’s syndrome was retracted. Neuropsychological tests showed cognition disorders concerning short- and long-term memory, feelings of depression, fear, paranoia and anger, agitation, loss of concentration and initiative, and difficulties with divided attention. Over the years, his symptoms worsened, with increasing cognitive problems, ataxia, tremor, hallucinations and dependency on others for his personal care and hygiene. He was well aware of his deterioration and started a euthanasia trajectory and passed away at 65 years. The disease course of both donors is summarized in Fig. 1A.

### MRI findings

Both donors showed general athrophy throughout the cortex (Fig. 1B, C, F and G) and dilation of ventricles (Fig. 1C, E and G). White matter intensity changes were observed around the ventricles, and small lesions were observed (Fig. 1B, C, D, E and M Donor 1 (Fig. 1E) and Donor 2, (Fig. 1K). While Donor 2 suffered from a stroke 10 years prior to his passing, a susceptibility weighted imaging for Donor 2 revealed no large vascular damage (Fig. 1L). No obvious thinning of the corpus callosum was seen (Fig. 1M). Overall, both donors revealed mild abnormalities on MRI including periventricular white matter intensities and mild global athrophy fitting with several neurodegenerative diseases.

### P62-inclusion pathology

**Figure 2:**
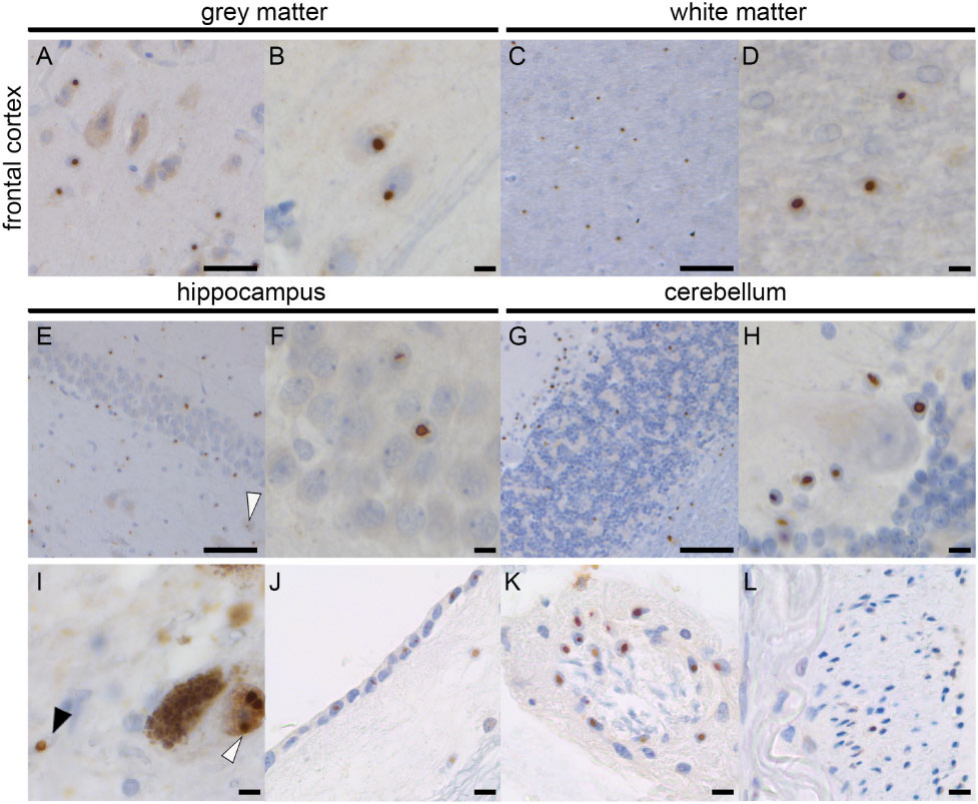
**p62-Pathology in Donor 2.** Abundant p62-positive nuclear inclusions in the frontal cortex grey matter (**A**, **B**) and white matter (**C**, **D**). The hippocampus shows nuclear inclusions in the CA4 neuron (white arrowhead) and the dentate gyrus (**E**, **F**). In the cerebellum, inclusions are seen in the white matter and granular layer (**G**, **H**). No inclusions were seen in the Purkinje cells, whereas most Bergmann glia showed inclusions (**H**). Pathology was also seen in the dopaminergic neurons (white arrowhead) and astrocytes (black arrowhead) in the substantia nigra (**I**), ependymal (**J**), extra-axial cells of the third nerve (**K**) and choroid plexus cells (**L**). Scale bar **A** is 50 μm; Scale bar **B**, **D**, **F**, **H**, **I**–**L** is 10 μm; scale bar **C**, **E**, **G** is 100 μm.

Intranuclear p62-positive inclusions were found in all brain structures, similar as reported before for FXTAS (Greco *et al.*, 2006). Cortical structures such as the frontal cortex showed inclusions in neurons and astrocytes in grey and white matter (Fig. 2A–D). Hippocampus showed many inclusions in CA and dentate gyrus granular cells (Fig. 2E and F). In the cerebellum, inclusions were observed in the white matter and granular cell layer, and in most of the Bergmann glia but not in the Purkinje cells (Fig. 2G and H). The substantia nigra (SN) showed inclusions in astrocytes and dopaminergic neurons (Fig. 2I). Similar to earlier reported findings in FXTAS, inclusions were also found in the ependymal layer (Fig. 2J) and choroid plexus (Fig. 2K). Additional findings include the presence of inclusions in the extra-axial part of nerve 3, but not intra-axial (Fig. 2L).

### Vascular pathology

**Figure 3:**
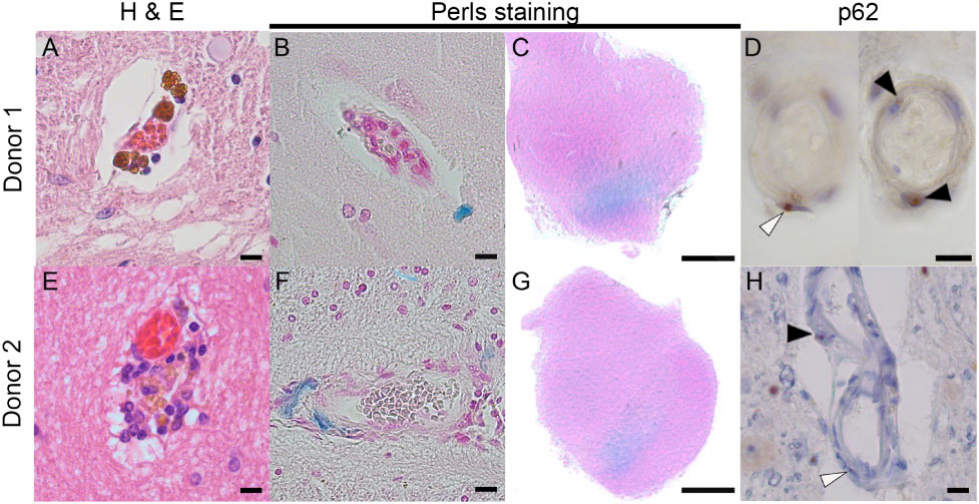
**Vascular pathology of Donors 1 and 2.** Brown iron-like deposits are seen in both donors (**A**, **E**), which stain blue in a Perls staining (**B**, **F**). Both donors showed abnormal increased iron deposits in the ventral part of the substantia nigra (**C**, **G**). Throughout the brain, inclusions were seen in the endothelial cells (black arrowhead) and the pericytes (white arrowhead) (**D**, **H**). Scale bar **A**, **B**, **D**, **E**, **F**, **H** is 10 µm; scale bar **C**, **G** is 0.5 cm.

Both donors showed vascular lesions such as lacunae and strokes throughout the brain. In the H&E staining, brown iron deposits were appreciated (Fig. 3A and E). Perls staining showed that the disrupted vasculature resulted in iron deposits, found throughout the cortex and hippocampus in normal age-related amounts (Fig. 3B and F), and an abnormal increased amount of iron deposits in the ventral region of the SN (Fig. 3C and G). Small p62-positive intranuclear inclusions were observed in the endothelial cells of the blood vessels, as well as in the pericytes (Fig. 3D and H). An overview of p62-inclusions in different cell types and iron deposits visualized by Perls staining is provided per region in Table 1.

**Figure 4:**
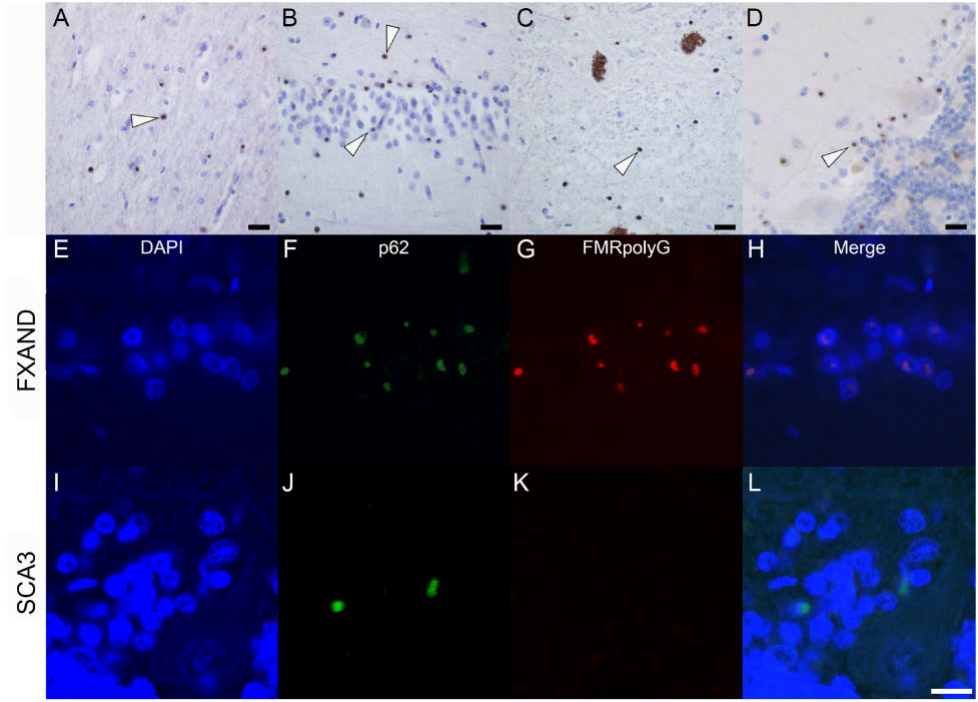
**Figure 4 FMRpolyG-positivity in Donor 2.** FMRpolyG (1C5)-positive inclusions are seen in the frontal cortex (**A**), hippocampus (**B**), substantia nigra (**C**) and cerebellum (**D**). Inclusions are indicated with white arrowheads. Co-localization of p62 and FMRpolyG (NTF1) immunopositivity in the Bergmann glia of the cerebellum in Donor 2 and spinocerebellar ataxia 3 (SCA3) donor. Bergmann glia shown with DAPI (**E**, **I**) showed inclusions with p62 (**F**, **J**) and FMRpolyG (**G**, **K**). Co-localization of the immunopositivity shows overlap in FXAND (**H**) and not in SCA3 (**L**). Scale bar is 10 μm.

**Table 1:** Distribution and burden of inclusion pathology and iron deposits throughout the brain of Donors 1 and 2

		Donor 1				Donor 2			
		p62				p62			
Area	Region	Neuron	Astrocytes	Vessel	Perls	Neuron	Astrocytes	Vessel	Perls
**Cortex**	Frontal cortex	•••	•••	••	•	•••	•••	•••	••
	Temporal cortex	•••	•••	••	•	•••	•••	••	••
	Parietal	••	••	•	•	•••	•••	••	•
	Occipital cortex	••	••	•	•	••	•••	••	•
	Insular cortex	••	•••	•	•	••	•••	•	•
**Limbic**	Amygdala	••	••	•	–	••	•••	–	–
	Hippocampus	•••	•••	••	–	•••	•••	•••	–
	Entorhinal cortex	•	••	•	•	••	•••	•	–
**Sub-cortical**	Putamen	•••	•••	•••	•••	••	••	•	•••
	Globus pallidus	•••	•••	•••	•••	•	••	•	•••
	Caudatus	•••	•••	••	•••	••	•••	••	•••
	Thalamus	••	••	•	••	••	••	•	•
**Cerebellum**	Dentate nucleus	•	••	•	–	•	•••	•	–
	Folia	–	•••	•	–	–	•••	–	–
**Brainstem**	Substantia nigra	••	••	•••	••	•••	•••	•••	•••
	Locus ceruleus	•••	•••	•	–	•••	•••	–	–

p62, not present; •, few inclusions present; ••, moderate amount of inclusions present; •••, abundant amount of inclusion present. Perls, not present; •, few iron deposits; ••, moderate amount of iron deposits; •••, abundant amount of iron deposits.

### FMRpolyG pathology

FMRpolyG was detected in all regions of the brain. FMRpolyG intranuclear inclusions are shown in the frontal cortex (Fig. 4A), the hippocampus (Fig. 4B), the astrocytes in SN (Fig. 4C) and in the Bergmann glia in the cerebellum (Fig. 4D). FMRpolyG positivity was also observed in the vasculature. Double-immunofluorescence for FMRpolyG and p62 showed that FMRpolyG protein co-localizes with p62 inclusions in the cerebellum (Fig. 4E–H), where an occasional p62-inclusion did not show FMRpolyG positivity. As a negative control, no immunoreactivity of FMRpolyG was observed in a case of spinocerebellar ataxia 3 (SCA3) (Fig. 4I–L), confirming the specificity of our antibodies for the *FMR1*-premutation-translated product, FMRpolyG.

## Discussion

Here, we report two cases with dementia and psychiatric symptoms who were diagnosed as *FMR1*-premutation carriers either post-mortem or in advanced disease state. While initial symptoms suggested dementia, both donors showed mild movement disturbances during the progression of the disease. MRI did not reveal the characteristic FXTAS-related imaging profile, which, in combination with the symptoms, posed a challenge for the attending clinicians to diagnose the patients as potential *FMR1*-premutation carriers. Interestingly, both donors experienced cerebrovascular accidents which could be explained by the post-mortem findings of FMRpolyG inclusions in the endothelial cells and pericytes. We propose that these brain vascular lesions may contribute to the complex symptomology of *FMR1*-premutation carriers.

### Clinical findings

The clinical phenotype for FXTAS has been described in detail, where movement symptoms are the most prominent features of the disease (Hagerman, 2013; Leehey *et al.*, 2016; Robertson *et al.*, 2016). However, the two *FMR1*-premutation individuals described here exhibited only mild movement symptoms such as rigidity and bradykinesia, and showed predominantly psychiatric problems, matching the recent description of FXAND (Hagerman *et al.*, 2018). Interestingly, Donor 1 experienced polyneuropathy as one of the first symptoms, which can be a prevalent sign in *FMR1*-premutation carriers with FXTAS presentation (Hagerman *et al.*, 2007; Berry-Kravis *et al.*, 2007b). Of clinical importance, the two donors did not show the typical middle cerebral peduncle imaging feature of FXTAS; however, they did show mild atrophy, similar to other dementias. This study highlights the challenge for clinicians to diagnose *FMR1*-premutation carriers, and stresses the importance of genetic screening for CGG repeats within *FMR1* for suspected dementia with behavioural features.

### Inclusion pathology

The p62-inclusion pathology of the two cases described here shows a similar distribution pattern as described in FXTAS (Tassone, 2004; Greco *et al.*, 2006). However, we describe here an additional feature with inclusions present in the endothelial cells and pericytes that can compromise vascular function. As both donors also suffered from multiple vascular incidents in the brain, this suggests that apart from cellular dysfunction, problems in the brain vasculature can contribute to the *FMR1*-premutation phenotype where infarcts or transient ischaemic attacks can disrupt blood supply. Consistent with our hypothesis, a high incidence of cardiac abnormalities and cerebrovascular disease has been observed in FXTAS donors (Hall *et al.*, 2005; Hamlin *et al.*, 2012), which could be linked to a decline in vascular integrity due to inclusion pathology. In addition, inclusions have been observed in cardiomyocytes (Hunsaker *et al.*, 2011), suggesting that the peripheral vasculature could also be compromised. Furthermore, inclusions were found in the extra-axial part of the nerve sheet, it is possible that the neuropathy can be explained by nerve involvement in the sensory nerves, which has to be studied in future autopsies of carriers.

## Conclusion

Our study also confirms that the inclusion pathology in carriers of the *FMR1* premutation can be visualized with antibodies directed against FMRpolyG (Krans *et al.*, 2019), with almost all p62-positive inclusions also positive for FMRpolyG. However, we noted that the dopaminergic neurons of the SN showed multiple p62-positive inclusions in one cell that were not positive for FMRpolyG. These inclusions are most likely Marinesco bodies (Beach *et al.*, 2004), which are age-related nuclear inclusions restricted to the SN. FMRpolyG labelling is specific to the protein translated from the *FMR1*-premutation, and it is not present in the polyQ aggregates found in SCA3. Importantly, we propose that FMRpolyG antibodies can be used as diagnostic tools to identify *FMR1*-premutation carriers post-mortem, notably in large retrospective brain bank cohorts.

## Abbreviations

FXAND =: Fragile X-associated neuropsychiatric disorders
FXPOI =: Fragile X-associated primary ovarian insufficiency
FXTAS =: Fragile X-associated tremor/ataxia syndrome
H&E =: haematoxylin and eosin
NBB =: Netherlands Brain Bank
RAN =: repeat-associated non-AUG-initiated
SCA3 =: spinocerebellar ataxia type 3
SN =: substantia nigra
